# Delayed-onset hearing loss in first-grade students who previously passed the newborn hearing screening

**DOI:** 10.3389/fped.2025.1623225

**Published:** 2025-08-21

**Authors:** Reem Elbeltagy, Waad AlObayed, Shatha Mashbri, Robina Alrasheed, Renad Albakiri, Munira Almulayfi, Rania Alkahtani

**Affiliations:** Department of Health Communication Sciences, College of Health and Rehabilitation Sciences, Princess Nourah bint Abdulrahman University, Riyadh, Saudi Arabia

**Keywords:** hearing, screening, school, children, PTA, tympanometry, OAE = otoacoustic emission

## Abstract

**Introduction:**

Newborn hearing screening is essential for the early detection of hearing loss, enabling timely intervention that supports communication and academic success. However, some children may develop delayed-onset hearing loss, which can go undetected without ongoing monitoring. Even mild hearing loss can affect educational development, highlighting the importance of preschool hearing screening. This study aimed to investigate the prevalence of delayed-onset hearing loss in first-grade students who had previously passed newborn hearing screening, emphasizing the need for early identification.

**Methods:**

A cross-sectional study was conducted involving 130 first-grade students. The screening protocol included otoscopic examination, Pure Tone Audiometry (PTA), tympanometry, and Transient Evoked Otoacoustic Emissions (TEOAE). Passing criteria were defined as PTA thresholds ≤20 dBHL at 500–4,000 Hz, Type A or Ad tympanogram, and a TEOAE signal-to-noise ratio ≥3 dB.

**Results:**

Of the participants, 80 (61.5%) passed the hearing screening, while 50 (38.5%) failed. Among those who failed, 43 (86%) showed abnormal tympanometry results, indicating potential conductive hearing loss, whereas 7 (14%) failed both OAE and PTA despite having normal tympanometry and were confirmed to have sensorineural hearing loss (SNHL). The prevalence of delayed-onset SNHL was therefore 5.4%. Among the risk factors examined, consanguinity was significantly associated with the presence of hearing loss.

**Conclusion:**

The observed 5.4% prevalence of delayed-onset sensorineural hearing loss among first-grade students underscores the importance of implementing preschool hearing screening to ensure early detection and timely intervention.

## 1 Introduction

Hearing loss is the most common sensory disorder in humans, with a prevalence higher than that of diabetes, vision impairments, or musculoskeletal abnormalities (1). Reported prevalence rates vary widely due to differences in definitions and criteria used to classify hearing loss across studies. Data on prevalence, incidence, and severity are essential for estimating the social, emotional, physical, and financial impacts of hearing loss (1). The World Health Organization (WHO) reported that 34 million children worldwide live with hearing loss (2). The global economic burden of untreated hearing loss is estimated at USD 980 billion annually, encompassing healthcare, education, productivity losses, and societal costs (2). While often associated with aging, hearing loss also affects a significant number of children and adolescents, carrying significant negative consequences for speech and language development, social-emotional well-being, and, critically, academic performance (3, 4).

While newborn hearing screening (NHS) programs are highly effective in identifying congenital hearing loss, they may not detect all forms of hearing impairment (5, 6). A subset of children may develop delayed-onset sensorineural hearing loss (SNHL), which emerges after a successful newborn screening and can progressively worsen over time. Delayed-onset SNHL can be caused by a variety of factors, including progressive genetic syndromes, ototoxic medication exposure, or complications from infections such as cytomegalovirus or bacterial meningitis. Without targeted follow-up, this late-emerging hearing loss can remain undetected for years, leading to a silent and cumulative impact on development (6, 7). This poses a significant diagnostic and management challenge, as these children are often perceived to be at low risk and may not receive ongoing auditory monitoring. In particular, parents may face difficulties in seeking a diagnosis for their children (8, 9).

To bridge the diagnostic gap between the NHS and the age when hearing loss is often clinically confirmed in children with delayed-onset SNHL, it is recommended to conduct hearing screenings at school-age (10, 11). Even unilateral or minimal hearing loss can negatively impact educational performance and social wellbeing (4, 11–14). Children may be misidentified as inattentive or slow learners, which can adversely affect academic performance, peer relationships, self-esteem and mental health (10, 15, 16). Hearing loss interferes with classroom engagement, comprehension, and participation, limiting both academic success and future opportunities for employment and social integration (11, 17). According to the Office of Special Education Programs, 1% of students receiving special education services in 2020 were categorized as having a hearing impairment (18). Early detection is therefore essential to improve educational and social outcomes for affected children.

By first grade, children undergo a critical transition from pre-literacy foundations to structured academic instruction that includes decoding, reading fluency, writing, and complex verbal comprehension. At this stage, instruction becomes explicit and systematic, focusing on phonics, word recognition, and comprehension strategies essential for literacy development (19). Efficient decoding skills acquired in early grade levels directly support reading fluency and later reading comprehension (20). This transition to formal learning heavily depends on the integrity of auditory abilities. Children with intact hearing and auditory discrimination skills are better equipped to perceive subtle linguistic cues, which are crucial for phoneme awareness and segmentation, key predictors of literacy success (21). In contrast, children with even mild hearing loss may demonstrate delays in oral language and phonological processing, which can negatively impact their academic performance (22). These findings highlight the importance of early identification and support for auditory-related difficulties to ensure that all children can access and benefit from foundational literacy instruction.

Therefore, this pilot study aimed to generate preliminary data on the prevalence of delayed-onset SNHL among first-grade students who had passed the newborn hearing screening. Identifying delayed-onset SNHL at this specific juncture allows for timely and effective intervention, as it represents a window of opportunity where educational and therapeutic support can still mitigate long-term academic deficits and prevent a widening learning gap.

## 2 Materials and methods

### 2.1 Study design and setting

A cross-sectional pilot study was conducted in elementary schools within the Riyadh region of Saudi Arabia. Three private schools were selected through convenience sampling based on their willingness to participate and their geographic distribution across the northern, eastern, and western parts of the city, to allow for some socioeconomic variation. No formal sample size calculation was conducted, as the aim was to explore feasibility and generate preliminary data to inform the design of future large-scale studies.

### 2.2 Participant recruitment and parental questionnaire

Parents were invited to complete an online questionnaire to provide information regarding their child's hearing history. Specifically, they were asked whether their child's hearing had been screened at birth and, if so, whether the child passed the screening. The questionnaire did not inquire about the type of hearing test administered at birth (e.g., otoacoustic emissions or automated auditory brainstem response), nor did it differentiate between well-baby and newborn intensive care unit (NICU) screenings. All information regarding NHS was based solely on parental report, and no medical records were reviewed to confirm screening modality or neonatal status. Additionally, parents were asked to report on potential risk factors for hearing loss in their child, such as family history, parental consanguinity, medical conditions, or experiencing recent flu (see Supplementary Appendix A). All information provided was based solely on parental report. This reliance on retrospective parental reporting represents a limitation, as it may introduce recall bias and inaccuracies, particularly regarding screening details or risk factors that occurred several years prior.

### 2.3 Inclusion and exclusion criteria

#### 2.3.1 Inclusion criteria

- First-grade students whose parents provided consent for participation in the hearing screening conducted for the purpose of this study.
- Students reported by their parents to have passed the newborn hearing screening.

#### 2.3.2 Exclusion criteria

- Students who were not screened for hearing loss at birth.
- Students who failed the newborn hearing screening.
- Students currently using hearing aids or cochlear implants, indicating a previously confirmed diagnosis of hearing loss.
- Students with impacted cerumen at the time of screening, as this could compromise the accuracy of hearing test results.

### 2.4 Hearing screening procedures

According to ASHA and AAA (10, 11), Pure Tone Audiometry (PTA), Tympanometry and Otoacoustic Emissions (OAE) are appropriate screening tools for school-aged children. In the current study, the following procedures were used for hearing screening:

- Otoscopic Examination (Otoscope: Avondale): conducted to identify abnormalities in the external auditory canal or tympanic membrane.
- Pure Tone Audiometry (PTA) (Amplivox Diagnostic Audiometer 240): Screening was performed at 20 dB HL at 0.5, 1, 2, and 4 kHz using air conduction. A pass required a positive response at all frequencies in both ears (10).
- Tympanometry (Interacoustics): A Type A or Ad tympanogram was considered a pass. A Type B was classified as abnormal (flat), and a Type C tympanogram was considered abnormal if middle ear pressure was > 250-daPa, in accordance with ASHA guidelines (11).
- Transient Otoacoustic Emissions (TOAE) (Otodynamics): A pass was defined as a signal-to-noise ratio of ≥3 dB at frequencies ranging from 500 to 4,000 Hz (10).

### 2.5 Failing criteria and follow-up testing

For both PTA and TEOAE, failure was defined as a lack of response to any test frequency at screening levels in either ear, in accordance with ASHA guidelines (11). Specifically, for PTA, screening was conducted at fixed presentation levels of 20 dB HL at 0.5, 1, 2, and 4 kHz. If a child did not respond to any of these frequencies in either ear, the result was considered a failure. For TEOAE, screening was performed across 500 to 4,000 Hz. A pass required a signal-to-noise ratio of ≥3 dB at all four frequencies in each ear. If this criterion was not met in either ear, the result was classified as a failure.

- A failed result suggestive of SNHL was characterized by Type A or Ad tympanogram combined with a failure on PTA and/or TEOAE. Children who failed PTA and/or TEOAE but presented with a Type A or Ad tympanogram were further evaluated using both air and bone conduction testing at the school, in the same quiet room used for initial screening, to determine the type and degree of hearing loss. All follow-up testing was conducted by certified audiologists using calibrated equipment, and cases of SNHL were diagnosed based on standard audiological criteria. Hearing loss severity was classified according to ASHA guidelines, with mild hearing loss defined as thresholds between 26 and 40 dB HL, moderate as 41–55 dB HL, moderately severe as 56–70 dB HL, severe as 71–90 dB HL, and profound as greater than 90 dB HL (23).
- A failed result suggestive of potential conductive hearing loss (CHL) was characterized by a flat tympanogram (Type B) or abnormal middle ear pressure (Type C tympanogram) in accordance with ASHA guidelines (11). Participants with a Type C tympanogram were included in this group, as Eustachian tube dysfunction, reflected by a Type C pattern, can cause minimal or mild CHL in some cases (24). Sente and Sente (25) reported that Eustachian tube dysfunction may result in CHL, with hearing loss not exceeding 25 dB.

### 2.6 Testing environment and child engagement

All screening and diagnostic tests were conducted by audiologists in a quiet room within the school premises. Ambient noise levels were monitored using a sound level meter (Norsonic Nor132) and maintained below 45 dBA to ensure testing accuracy and the reliability of the measurement. During PTA, children were instructed to raise their hand upon hearing any tone, even very soft ones. To encourage participation and maintain engagement, children were rewarded with stickers featuring their favorite cartoon characters as a form of positive reinforcement.

### 2.7 Ethical consideration

The study was approved by the Institutional Review Board of Princess Nourah bint Abdulrahman University (IRB Log Number: 23-0750). Informed consent was obtained from the parents prior to data collection. Participation was voluntary, and verbal assent was obtained from the children before testing.

### 2.8 Statistical analysis

Data were analyzed using the Statistical Package for the Social Sciences (SPSS). Descriptive statistics, including frequencies and percentages, were used to summarize the data. Additionally, the Chi-square test was employed to examine associations relevant to the study objectives. Logistic regression analysis was conducted to estimate the magnitude of the association between identified risk factors and the outcome. Statistical significance was determined at a *p*-value of ≤0.05.

## 3 Results

A total of 196 parents responded to the questionnaire. Of these, 57 reported that their child either had not undergone newborn hearing screening, were unsure whether the screening had been performed, or indicated that the child had failed the screening. These cases were excluded from the study. Consequently, 139 children were scheduled for hearing assessment. Upon examination, 9 children were excluded due to excessive cerumen impaction that prevented accurate testing. The final sample consisted of 130 children, including 39 males (30%) and 91 females (70%).

Table 1 summarizes participant background characteristics and potential risk factors for hearing loss as reported by parents. The majority of children were born full term (96.9%), had no recent health symptoms (72.3%) or history of ear infections (96.2%), and did not have a family history of hearing loss (91.5%). Most parents reported no consanguinity (75.4%) or maternal health issues during pregnancy (94.6%), and childbirth complications were rare (5.4%).

**Table 1:** Parental reports of participant potential risk factors for hearing loss.

Risk factor	Parental response	N	%
Birth maturity	Full term	126	96.9%
	Premature	4	3.1%
Recent health symptoms	No symptoms	94	72.3%
	Cold/sore throat/fever/cough	36	27.7%
History of ear infections	No	125	96.2%
	Yes	5	3.8%
Family history of hearing loss	No	119	91.5%
	Yes	11	8.5%
Parental consanguinity	Not related	98	75.4%
	Related	32	24.6%
Prenatal health issues	No health issues	123	94.6%
	Health issues reported	7	5.4%
Childbirth complications	None	123	94.6%
	Present	7	5.4%

**Figure 1:**
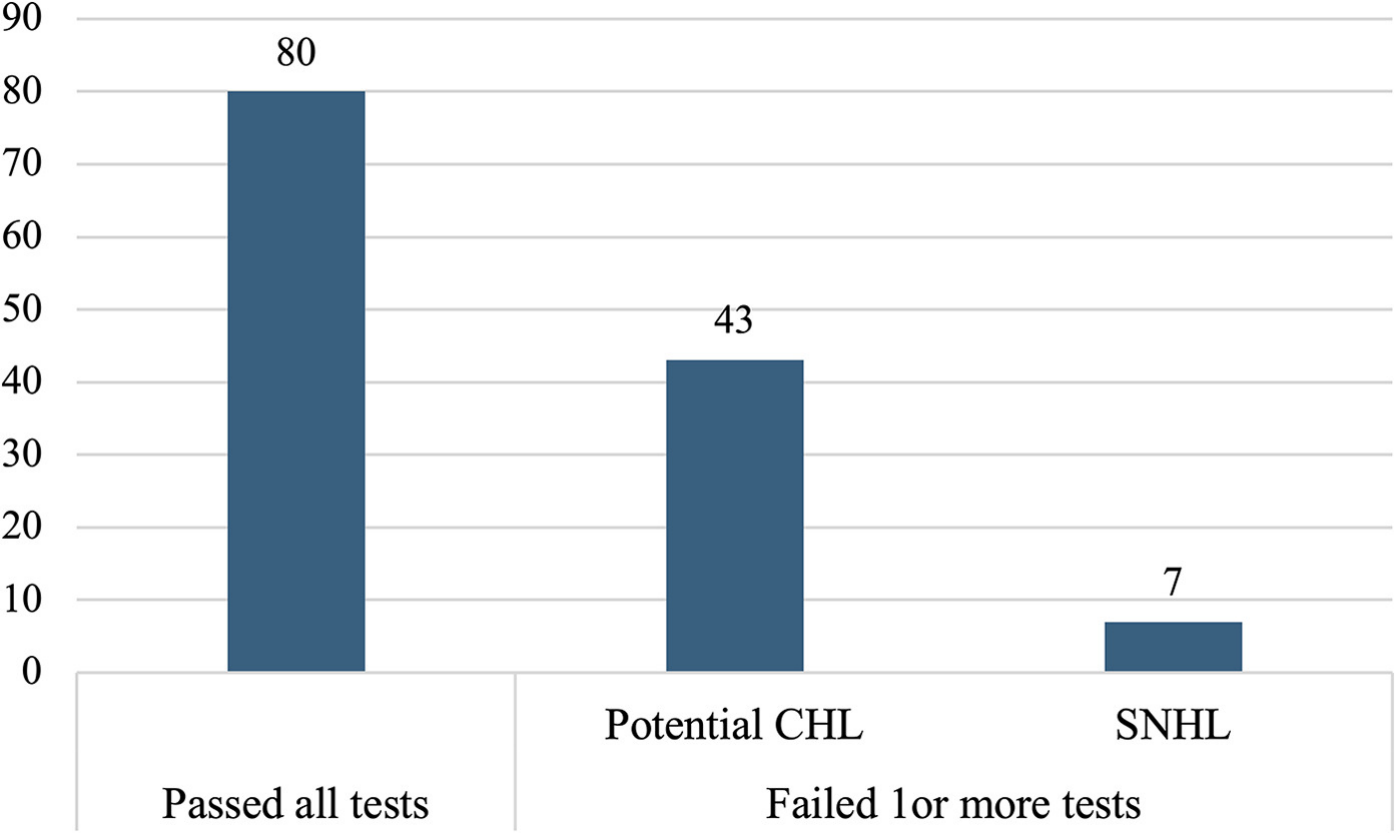
Hearing screening outcomes and types of hearing loss. Children with potential CHL were identified based on tympanometry, while SNHL was confirmed through air- and bone-conduction audiometry.

**Figure 2:**
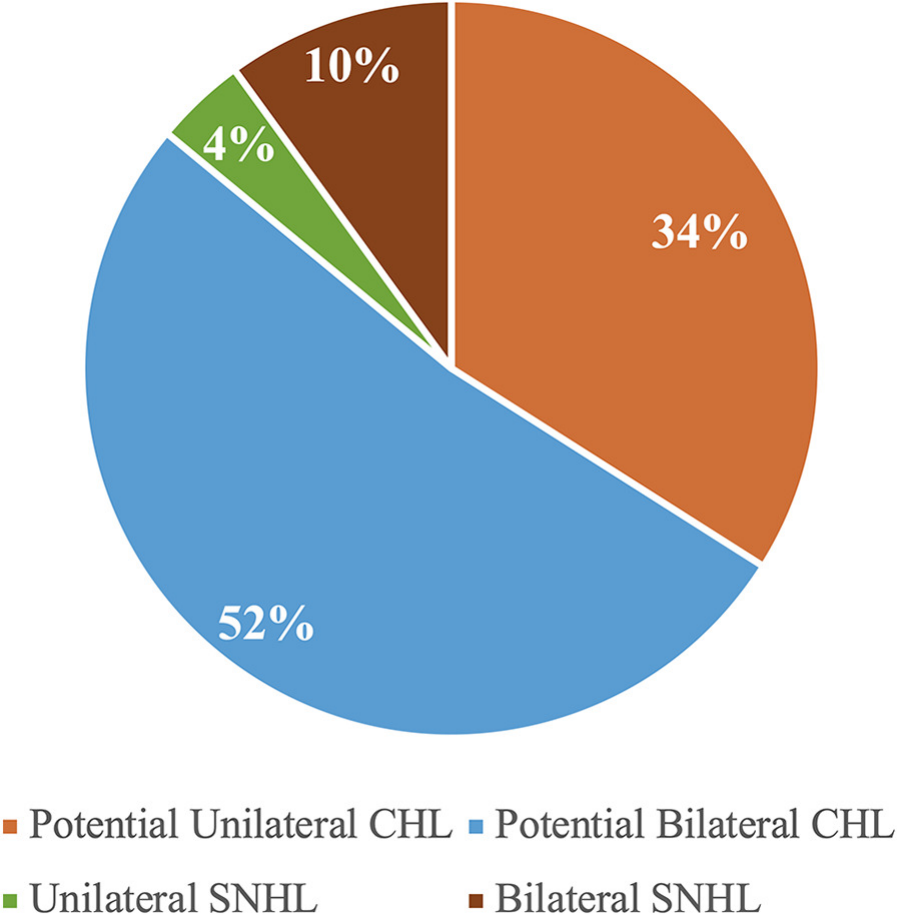
Distribution of laterality and type of hearing loss among children who failed the screening (*n* = 52).

The outcomes of the hearing screening tests conducted at the current study along with the types of hearing loss identified in the participants who failed at least one of the screening tests are illustrated in Figure 1. As it appears, majority of the participants (*n* = 80, 61.5%) passed all the three screening tests, while 50 (38.5%) failed in at least one test. Of the 50 participants who failed, 43 (86%) were identified with potential CHL, while 7 (14%) had a confirmed SNHL. Table 2 shows the outcomes of each screening test and Figure 2 shows the laterality of the hearing loss of all participants who failed the screening tests. The characteristics of the seven participants identified with SNHL are presented in Tables 3, 4.

**Table 2:** Outcomes of tympanometry, PTA, and OAE screening among participants (*n* = 130).

Test	Category		N		%	
Tympanometry	Fail	Type B	31	Total = 43	23.8%	33%
		Type C	12		9.2%	
	Pass (Type A and/or Ad)		85		63.4%	
**PTA**	Fail		13		10.0%	
	Pass		117		90.0%	
**OAE**	Fail		32		24.6%	
	Pass		98		75.4%	

Failure refers to a lack of response in at least one ear. Tympanometry failure is based on Type B or Type C results.

**Table 3:** Risk factors, screening and diagnostic test outcomes of children identified with SNHL (*n* = 7).

Risk Factor		Child 1	Child 2	Child 3	Child 4	Child 5	Child 6	Child 7
Premature		No	No	No	No	No	No	No
Recent cold (runny nose), sore throat, fever, or cough		No	Yes	Yes	No	No	No	No
Ear infection		No	No	No	Yes	No	No	No
Family history of hearing loss		No	No	No	No	Yes	Yes	No
Parental consanguinity		No	No	Yes	No	Yes	No	Yes
Prenatal health issues		No	Yes	No	No	No	No	No
Postnatal health issues		No	No	No	No	No	No	No
Screening Tests	Tympanometry	A	Right: A Left: Ad	A	A	A	A	A
	PTA (Bilateral)	Fail	Fail	Fail	Pass	Fail	Pass	Pass
	OAE (Bilateral)	Pass	Fail	Fail	Fail	Pass	Fail	Fail
Degree of Hearing Loss		Mild	Mild	Mild to moderate	Mild	Mild	Mild	Mild

**Table 4:** Audiological profiles showing air conduction thresholds across frequencies for children identified with SNHL (*n* = 7).

Participant	Right ear^a^						Left ear^a^					
	250 Hz	500 Hz	1 kHz	2 kHz	4 kHz	8 kHz	250 Hz	500 Hz	1 kHz	2 kHz	4 kHz	8 kHz
Child 1	20	20	25	30	35	30	25	25	30	25	30	30
Child 2	15	15	30	35	20	25	15	20	15	15	20	20
Child 3	25	40	45	45	45	40	30	40	50	40	40	45
Child 4	25	30	25	35	35	30	25	25	15	35	30	35
Child 5	10	15	15	20	20	20	25	20	25	35	35	30
Child 6	15	20	35	25	35	30	15	25	15	30	30	35
Child 7	25	25	30	30	25	25	20	25	25	40	30	35

^a^ Air-bone gab was ≤10 dB for all children.

Bivariate analysis using the Chi-square test showed a statistically significant association between parental consanguinity and the presence of hearing loss among children (*χ*^2^ = 3.86, *p* = 0.05) (Table 5). To further examine this relationship while adjusting for other potential risk factors, a binary logistic regression analysis was conducted. The regression model included family history of hearing loss, parental consanguinity, prenatal and postnatal health issues, recent cold, history of ear infection, and maturity at birth. Among all predictors, only parental consanguinity remained significantly associated with hearing loss (*p* = 0.026) (Table 6).

**Table 5:** Association between risk factors and presence of hearing loss based on chi-square analysis.

Risk factor	Parental response	N (%)	Fail (*N* = 50)	Pass (*N* = 80)	X^2^	*P*-Value
Premature	Yes	n	2	2	0.232	0.630
		%	50.0%	50.0%		
	No	n	48	78		
		%	38.1%	61.9%		
Recent cold (runny nose), sore throat, fever, or cough	Yes	n	18	18	2.80	0.094
		%	50.0%	50.0%		
	No	n	32	62		
		%	34%	66%		
History of ear infection	Yes	n	3	2	1.02	0.313
		%	60.0%	40.0%		
	No	n	47	78		
		%	37.6%	62.4%		
Family history of hearing loss	Yes	n	3	8	0.636	0.425
		%	27.3%	72.7%		
	No	n	47	72		
		%	39.5%	60.5%		
Parental consanguinity	Yes	n	17	15	3.86	0.050^a^
		%	53.1%	46.9%		
	No	n	33	65		
		%	33.7%	66.3%		
Prenatal health issues	Yes	n	4	3	1.09	0.296
		%	57.1%	42.9%		
	No	n	46	77		
		%	37.4%	62.6%		
Postnatal health issues	Yes	n	2	5	0.306	0.580
		%	28.6%	71.4%		
	No	n	48	75		
		%	39.0%	61.0%		

^a^ Statistically significant at ≤0.05.

**Table 6:** Binary logistic regression analysis of risk factors associated with the presence of hearing loss.

Risk factor	Odd ratio	95% CI^a^	*P*-value
Family history	.611	0.18–2.03	.422
Parental consanguinity	2.620	1.12–6.10	.026^b^
Prenatal health issues	1.363	0.22–8.42	.739
Postnatal health issues	1.433	0.29–7.04	.658
Recent cold	.676	0.29–1.59	.370
Ear infection	.449	0.07–3.08	.415
Birth maturity	1.646	0.20–13.35	.641

^a^ CI: confidence interval.

^b^ Statistically significant at ≤0.05.

## 4 Discussion

This study aimed to determine the prevalence of delayed-onset SNHL in first-grade students who reportedly passed newborn hearing screening per parental report. Hearing status was assessed using four procedures: otoscopic examination, PTA, tympanometry, and OAE. These procedures were selected in accordance with the recommendations of ASHA and AAA for screening school-age children (10, 11).

In the current study, 50 (38.5%) participants failed the screening, while 80 (61.5%) passed (Figure 1), a notably high failure rate. Globally, the reported prevalence of hearing loss among school-aged children varies widely, from 0.88% to 46.7% (26). The elevated failure rate in this study falls within this range, likely due to methodological and demographic factors. For example, Skarżyński et al. (27) reported a screening failure rate of 16.4% among children aged 6–13 years, with younger children exhibiting higher failure rates. The high failure rate observed here may partly reflect this age effect, as first-graders are younger and more susceptible to middle-ear conditions, as evidenced by the large number of participants with Type B tympanograms (Table 2). Additionally, Altas et al. (28) reported a lower failure rate (27.2%), but their screening protocol included only PTA, without tympanometry or OAE testing, which may explain the differences between the two studies.

An increased prevalence of Type B tympanograms (23.8%) was observed in the current study, indicating a high incidence of otitis media. This aligns with Al-Rowaily's (29) findings of frequent otitis media in Saudi children, and Westerberg's (30) identification of otitis media as a common cause of school-age hearing loss. The higher prevalence of potential CHL compared to SNHL observed in this study is not unexpected, as CHL resulting from otitis media is common among young children (31–34). Previous studies conducted in Saudi Arabia have also reported a higher prevalence of CHL compared to SNHL (29, 35, 36). The consistently high rate of CHL may reflect a widespread occurrence of middle ear disorders, possibly associated with seasonal respiratory infections or environmental allergens prevalent among young children (37). Confounding factors, such as seasonal middle-ear infections and environmental allergens, may also have contributed to the high prevalence of CHL, complicating the distinction between temporary conductive conditions and true sensorineural deficits. However, variations in tympanometric findings are evident across studies. For example, a study conducted in Brazil reported a lower prevalence of Type B tympanograms (38), whereas a study from Ahwaz, Iran, reported higher rates (39). Similarly, the prevalence of Type C tympanograms varied, being very low in some studies (39, 40) but higher in others, such as those by Alothman (35) and Tamanini (4). These discrepancies may reflect regional or environmental factors influencing middle-ear pathology and underscore the importance of localized public health interventions. Nevertheless, the primary aim of the current study was not to identify CHL but rather to detect cases of SNHL among children who had previously passed newborn hearing screening.

The relatively high rate of SNHL (5.4%) observed in this study warrants further investigation, particularly since all children were reported by their parents to have passed newborn hearing screening. NHS alone is generally insufficient to identify all cases of SNHL. This is because NHS programs are primarily designed to detect bilateral moderate-to-profound hearing loss, and may therefore miss unilateral or mild hearing loss (<35 dB HL), as highlighted by the World Health Organization (41). In addition, some hereditary forms of SNHL do not present at birth but emerge later in childhood, making them undetectable by newborn screening. In our study, NHS results were reported by parents, and we did not have access to medical records confirming the specific screening protocols or whether risk-based follow-up procedures were implemented.

In Saudi Arabia, several gene mutations (e.g., GIPC3, ILDR1, W77R, MYO15A, TMC1, TMPRSS3, and DFNB67) have been identified in families and are associated with childhood hearing loss (42–44). A contributing factor may be the high rate of consanguinity in the country, which increases the risk of both congenital and delayed-onset hearing loss (45–47). As shown in Table 4, three of the seven students identified with SNHL were born to consanguineous parents. The current study confirmed a significant association between consanguinity and hearing loss, with children from consanguineous marriages being 2.6 times more likely to develop hearing loss. This finding aligns with existing literature demonstrating a strong relationship between consanguinity and hereditary hearing loss in Saudi Arabia (48–51). These results highlight the role of genetic risk factors in delayed-onset SNHL, particularly in populations with high rates of consanguinity.

Although the Joint Committee on Infant Hearing (52) identifies family history of hearing loss, prematurity, parental consanguinity, and prenatal or postnatal health issues as risk factors for childhood hearing loss, these factors did not emerge as statistically significant in the present study (Tables 5, 6). While a few children with hearing loss had a family history of hearing loss, the overall association was not significant based on both chi-square and logistic regression analyses. This may reflect the small number of confirmed SNHL cases or the influence of additional unmeasured environmental or genetic variables. It is also possible that some families may have underreported or been unaware of hearing loss in relatives, particularly in extended family members or those with mild or undiagnosed conditions, leading to potential misclassification and dilution of the observed association. Similarly, recent illnesses reported in a few children did not appear to have a strong association with hearing outcomes.

These findings reinforce that children who pass newborn hearing screening may still be at risk of developing delayed-onset or progressive SNHL. The results support the value of routine school-based hearing screenings as a secondary safety net, particularly in settings with high rates of genetic risk factors such as consanguinity. Continued monitoring beyond infancy and timely follow-up for at-risk groups are essential to ensure early detection and intervention.

### 4.1 Limitations

This study is a pilot investigation, and its relatively small sample size may limit the generalizability of the findings. To enhance the robustness of future research, studies with larger and more representative samples are needed. Additionally, the cross-sectional design of this study captured data at a single point in time, which restricts insights into the auditory health trajectory of the population. Longitudinal studies are recommended to monitor changes in hearing function over time and to evaluate the long-term effectiveness of interventions for delayed-onset hearing loss.

Moreover, the exclusive inclusion of private schools limits the generalizability of the results, as public schools were not represented. The study also relied on retrospective parental reporting to determine whether children had passed newborn hearing screening and to identify potential risk factors for hearing loss. This approach may introduce recall bias and inaccuracies, particularly for events that occurred several years prior, such as perinatal complications or early medical history. Additionally, some families may have been unaware of or reluctant to disclose hearing loss in relatives, especially in extended family members or those with mild or undiagnosed conditions, which could have led to underreporting of family history as a risk factor. Although hearing tests were conducted in a quiet schoolroom, the absence of a fully soundproof environment may have contributed to false positives, particularly in PTA results.

These limitations suggest that the reported prevalence rates should be interpreted with caution. Future studies should ensure standardized testing environments, include a broader range of schools, and cross-validate parental reports with medical records to improve data accuracy and reduce potential recall bias.

## 5 Conclusions

Although all study participants were reported by their parents to have passed newborn hearing screening, 5.4% were later identified with delayed-onset SNHL, emphasizing the need for continued auditory monitoring throughout early childhood. Parental consanguinity was also associated with an increased risk of hearing loss. These findings highlight the limitations of newborn hearing screening alone in detecting late-onset or progressive SNHL and reinforce the importance of ongoing monitoring and early intervention, particularly in high-risk populations.

## Key recommendations include

• Implementing routine school-age hearing screenings for early detection and timely management of delayed-onset hearing loss.
• Adopting a multifactorial approach that considers genetic, environmental, and health-related risk factors.
• Conducting future longitudinal studies with larger sample sizes to track hearing changes over time and better understand the natural progression and risk factors of delayed-onset hearing loss in Saudi Arabia.

By integrating these strategies into public health and educational systems, early identification and intervention can be enhanced, supporting improved academic performance, communication skills, and overall well-being among at-risk children.

## Data Availability

The raw data supporting the conclusions of this article will be made available by the authors, without undue reservation.
