# Supplementary material for: Delayed-onset hearing loss in first-grade students who previously passed the newborn hearing screening
Source: Front Pediatr. 2025 Aug 21;13:1623225. doi: 10.3389/fped.2025.1623225 (PMC12408585; doi:10.3389/fped.2025.1623225)
Supplement: Supplementary file 1 [file Datasheet1.pdf]

Parental questionnaire

1- Was your child tested for hearing loss at birth?
   - Yes
   - No
   - Not sure

2- If yes, did your child pass the newborn hearing test?
   - Yes, my child passed the newborn hearing screening
   - No, my child did not pass the newborn hearing screening
   - Not sure

3- Was your child born prematurely?
   - Yes, my baby was born prematurely
   - No, he/she was born full term

4- Did the mother have any health issues during pregnancy?
   - Yes
   - No

5- Did the child experience any health problems during or after birth?
   - Yes
   - No

6- Are the child's parents related (e.g., cousins)?
   - Yes
   - No

7- Does anyone in the family have hearing loss?
   - Yes
   - No

8- Has your child had an ear infection within the last year?
   - Yes
   - No

9- Has your child experienced any of the following symptoms in the past or current week: cold (runny nose), sore throat, fever, or cough?
   - Yes
   - No
